# Engaging and Educating Older Adults and Caregivers in Age-Friendly Healthcare

**DOI:** 10.1093/geroni/igaa057.2397

**Published:** 2020-12-16

**Authors:** Erin Emery-Tiburcio, Rani Snyder

## Abstract

As the Age-Friendly Health System initiative moves across the US and around the world, not only do health system staff require education about the 4Ms, but older adults, caregivers, and families need education. Engaging and empowering the community about the 4Ms can improve communication, clarify and improve adherence to treatment plans, and improve patient satisfaction. Many methods for engaging the community in age-friendly care are currently in development. Initiated by Health Resources and Services Administration (HRSA)-funded Geriatric Workforce Enhancement Programs (GWEPs), Community Catalyst is leading the co-design of Age-Friendly Health System materials with older adults and caregivers. Testing these materials across the country in diverse populations of older adults and caregivers will yield open-source documents for local adaptation. Rush University Medical Center is testing a method for identifying, engaging, educating, and providing health services for family caregivers of older adults. This unique program integrates with the Age-Friendly Health System efforts in addressing all 4Ms for caregivers. The Bronx Health Corps (BHC) was created by the New York University Hartford Institute of Geriatric Nursing to educate older adults in the community about health and health behaviors. BHC developed a method for engaging and educating older adults that is replicable in other communities. Baylor College of Medicine adapted and tested the Patient Priorities Care model to educate primary care providers about how to engage older adults in conversations about What Matters to them. Central to the Age-Friendly movement, John A. Hartford Foundation leadership will discuss the implications of this important work.

